# Genetic analysis identifies potential transmission of low pathogenic avian influenza viruses between poultry farms

**DOI:** 10.1111/tbed.13199

**Published:** 2019-04-25

**Authors:** Saskia A. Bergervoet, Rene Heutink, Ruth Bouwstra, Ron A.M. Fouchier, Nancy Beerens

**Affiliations:** ^1^ Department of Virology Wageningen Bioveterinary Research Lelystad The Netherlands; ^2^ Department of Viroscience Erasmus MC Rotterdam The Netherlandss; ^3^ GD Animal Health Service Deventer The Netherlands

**Keywords:** avian influenza virus, low pathogenic avian influenza, poultry, between‐farm transmission, genetic analysis

## Abstract

Poultry can become infected with low pathogenic avian influenza (LPAI) viruses via (in)direct contact with infected wild birds or by transmission of the virus between farms. This study combines routinely collected surveillance data with genetic analysis to assess the contribution of between‐farm transmission to the overall incidence of LPAI virus infections in poultry. Over a 10‐year surveillance period, we identified 35 potential cases of between‐farm transmission in the Netherlands, of which 10 formed geographical clusters. A total of 21 LPAI viruses were isolated from nine potential between‐farm transmission cases, which were further studied by genetic and epidemiological analysis. Whole genome sequence analysis identified close genetic links between infected farms in seven cases. The presence of identical deletions in the neuraminidase stalk region and minority variants provided additional indications of between‐farm transmission. Spatiotemporal analysis demonstrated that genetically closely related viruses were detected within a median time interval of 8 days, and the median distance between the infected farms was significantly shorter compared to farms infected with genetically distinct viruses (6.3 versus 69.0 km; *p* < 0.05). The results further suggest that between‐farm transmission was not restricted to holdings of the same poultry type and not related to the housing system. Although separate introductions from the wild bird reservoir cannot be excluded, our study indicates that between‐farm transmission occurred in seven of nine virologically analysed cases. Based on these findings, it is likely that between‐farm transmission contributes considerably to the incidence of LPAI virus infections in poultry.

## 1 INTRODUCTION

Avian influenza (AI) is a highly contagious viral disease that affects birds. Avian influenza viruses are widespread in wild waterfowl that form the natural reservoir of AI viruses (Stallknecht & Shane, 1988), and can occasionally be transmitted to commercial poultry. The viruses carry two surface glycoproteins, haemagglutinin (HA) and neuraminidase (NA), which are used for virus classification (Webster, Bean, Gorman, Chambers, & Kawaoka, 1992). In birds, 16 HA (H1‐H16) and 9 NA (N1‐N9) subtypes have been identified (Fouchier et al., 2005; Olsen et al., 2006).

Most AI viruses are low pathogenic avian influenza (LPAI) virus strains that cause subclinical infections in poultry (Webster & Rott, 1987). In some cases, mild respiratory disease, a reduction in egg production or low mortality is observed (Gonzales & Elbers, 2018). Avian influenza viruses of subtypes H5 and H7 pose the greatest threat to commercial poultry because of their potential to evolve into highly pathogenic avian influenza (HPAI) viruses. Highly pathogenic avian influenza viruses typically cause severe illness and high mortality in poultry (Webster & Rott, 1987), and some subtypes have been shown to also infect humans (Fouchier et al., 2004; Kruy, Buisson, & Buchy, 2008). Hence, surveillance programmes are implemented for the early detection of LPAI and HPAI viruses of subtypes H5 and H7, which are also known as notifiable AI strains. In the Netherlands, poultry farms are screened serologically for AI virus infections at least once a year (Bouwstra et al., 2017; Gonzales et al., 2010). In addition, virological testing is performed upon notification of AI suspicions based on clinical signs or the detection of antibodies against H5 or H7 subtyped viruses. Non‐notifiable LPAI virus infections are often considered to be of lower risk. However, their circulation in poultry may promote the emergence of influenza virus strains that have the ability to be transmitted efficiently among poultry and even humans (Li et al., 2014). Reassortment of these viruses with more pathogenic strains may have serious consequences for both animal and public health.

Although wild birds are considered the primary source of AI virus infections in poultry, flocks may also become infected by subsequent spread between farms. Recent HPAI outbreaks have demonstrated that AI viruses can spread rapidly between farms (Dargatz, Beam, Wainwright, & McCluskey, 2016; Guinat et al., 2018; Stegeman et al., 2004), leading to huge economic losses in the poultry industry. Sustained between‐farm transmission of LPAI viruses has also been observed in commercial poultry, for example, during LPAI outbreaks of subtypes H7N2 (1996–1998 and 2001–2002) in the United States (Akey, 2003; Davison, Eckroade, & Ziegler, 2003; Dunn et al., 2003; Ziegler, Davison, Acland, & Eckroade, 1999); H7N1 (1999 and 2000–2001), H7N3 (2002–2003 and 2004) and H5N2 (2010–2012) in poultry‐dense areas in Italy (Capua & Alexander, 2004; Capua, Mutinelli, Marangon, & Alexander, 2000; Mughini‐Gras et al., 2014); and recurrent outbreaks of H9N2 infections in Asia and the Middle East (late 1990s–present) (Capua & Alexander, 2004; Gu, Xu, Wang, & Liu, 2017).

Various routes of between‐farm transmission have been suggested, such as direct contact between poultry or indirect via the movement of persons (e.g. visitors, farm personnel), contaminated materials (e.g. farm equipment, clothing) or vectors (e.g. rodents, insects) between farms (Leibler, Carone, & Silbergeld, 2010; Thomas et al., 2005; Velkers, Blokhuis, Veldhuis Kroeze, & Burt, 2017; Vieira, Hofacre, Smith, & Cole, 2009; Wanaratana et al., 2013). Moreover, transmission over short distances may occur when the virus is dispersed into the environment via water, air or dust (Brown, Goekjian, Poulson, Valeika, & Stallknecht, 2009; Horm, Gutierrez, Sorn, & Buchy, 2012; Jonges et al., 2015; Spekreijse, Bouma, Koch, & Stegeman, 2013). Geographical clustering of infected farms implies the occurrence of transmission between neighbouring farms or separate introductions from the same environmental source (Boender et al., 2007; Mulatti, Bos, Busani, Nielen, & Marangon, 2010). However, the exact route of introduction into poultry often remains untraced and mechanisms underlying between‐farm spread of AI viruses are not clearly understood.

Genetic analysis has frequently been used to study the emergence, evolution and between‐farm transmission dynamics of HPAI viruses (Bataille, van der Meer, Stegeman, & Koch, 2011; Fusaro et al., 2016; Xu et al., 2016; Ypma et al., 2013). Similar studies for LPAI are limited by the lack of genetic information, in particular for non‐notifiable AI strains. Low pathogenic avian influenza virus infections may remain unnoticed or are not reported because the mild symptoms are thought to be caused by other poultry diseases (Elbers, Gorgievski‐Duijvesteijn, Zarafshani, & Koch, 2010). Therefore, LPAI viruses are primarily detected during routine serological screening without confirmation by virus detection. For this reason, the contribution of between‐farm transmission to the occurrence of LPAI virus infections in poultry is largely unknown.

This study combines routinely collected surveillance data with genetic analysis to assess the contribution of between‐farm transmission to the overall incidence of LPAI virus infections in poultry. We analysed 220 serological and virological detections of LPAI virus infections that occurred in commercial poultry in the Netherlands between 2006 and 2016, to identify potential between‐farm transmission cases. Spatial analysis was performed for each potential between‐farm transmission case separately to determine whether infected farms clustered geographically. Subsequently, whole genome sequence analysis was performed to determine the genetic relationship between viruses isolated from potential between‐farm transmission cases. Genetic analysis was combined with information regarding time, distance and poultry type to identify epidemiological variables associated with between‐farm transmission. Better understanding of LPAI virus transmission routes into poultry and between farms is important to control virus spread in an early stage.

## 2 MATERIALS AND METHODS

### 2.1 Ethical statement

Poultry blood and swab samples were collected as part of the national AI surveillance program in the Netherlands, which is carried out for detecting LPAI virus infections of H5 and H7 subtypes in poultry. Samples were taken by authorized veterinarians and sent to the laboratory for routine diagnosis of AI virus infections. Sampling of poultry was carried out in accordance with Council Directive 2005/94/EC of 20 December 2005 on European Union measures for the control of AI (EU, 2005) and regulation TRCJZ/2005/1411 of 7 June 2005 concerning the prevention, control and monitoring of infectious animal diseases, zoonoses and transmissible spongiform encephalopathies (TSEs). This study analyses the test results obtained in the surveillance program. No new samples were collected for this study specifically.

### 2.2 Study population

Samples were collected between January 2006 and September 2016. The study population involved 2,379 commercial poultry farms in the Netherlands, consisting of farms holding broiler chickens (46%), layer chickens (42%), chicken breeders (8%), turkeys (2%) and domestic ducks (2%), with 45,000 animals per farm on average, as registered in 2013 with moderate fluctuations over the study period.

### 2.3 Serological monitoring

For serological monitoring, blood samples were collected from all commercial poultry farms in the Netherlands once a year, except outdoor layer chicken and turkey farms, which were sampled four times a year and each production cycle respectively. Screening of serum for the presence of influenza‐specific antibodies was performed by the GD Animal Health Service using the FlockChek AI MultiS‐Screen Ab Test Kit (IDEXX). Samples identified as positive for influenza‐specific antibodies were subsequently tested by the national reference laboratory Wageningen Bioveterinary Research (WBVR) in a H5 and H7 subtype‐specific haemagglutination inhibition (HI) test according to the OIE Manual of Standards for Diagnostic Tests and Vaccines (OIE World Organisation for Animal Health, 2015). If no antibodies against virus subtypes H5 or H7 were detected, the subtype specificity of the antibodies was determined using an in‐house protein microarray or a bead‐based multiplexed immunoassay of HA and NA antigens. Results were confirmed using influenza subtype‐specific HI tests, neuraminidase inhibition (NI) tests and NA‐specific enzyme‐linked immunosorbent assays (ELISAs) (OIE World Organisation for Animal Health, 2015).

### 2.4 Virological monitoring

Virological monitoring was performed to check for virus circulation upon detection of antibodies against H5 and H7 subtyped viruses or in case of clinical notification. Tracheal and cloacal swabs were collected by a specialist team of the Netherlands Food and Consumer Product Safety Authority (NVWA). These samples were analysed by WBVR using the real‐time reverse transcription polymerase chain reaction method targeting the matrix gene (M‐PCR) (Fouchier et al., 2000). Influenza virus‐positive samples were subsequently tested in a H5 and H7 subtype‐specific PCR (Slomka et al., 2007, 2009). The sequence of the HA proteolytic cleavage site was analysed to determine the pathogenicity of the virus (Gall, Hoffmann, Harder, Grund, & Beer, 2008). Amplified HA and NA gene fragments were analysed by Sanger sequencing to determine the virus subtype (Gall et al., 2008, 2009). To isolate viruses, swab samples were inoculated into the allantoic cavity specific‐pathogen‐free (SPF) embryonated chicken eggs (ECEs) (OIE World Organisation for Animal Health, 2015). Allantoic fluids positive for haemagglutination were characterized in a HI test using in‐house prepared antisera.

### 2.5 Sequencing

Whole genome sequences of LPAI viruses were generated by next‐generation sequencing (NGS), as described previously (Beerens et al., 2017). In short, RNA was purified from swab specimen or allantoic fluid using the High Pure Viral RNA Kit (Roche), amplified using universal primers and sequenced with a minimum sequence coverage of 1,000 reads using the paired‐end 200 Illumina MiSeq platform. Consensus sequences were generated in CLC Genomics Workbench (Qiagen) using a reference‐based method (Beerens et al., 2017) and submitted to GISAID's EpiFlu database (https://www.gisaid.org) (Shu & McCauley, 2017) (Table S1). A recent study identified a limit of 0.5% for reliable detection of minority variants in the influenza virus genome, based on the error rate of the NGS procedure (Van den Hoecke, Verhelst, Vuylsteke, & Saelens, 2015). In this study, we used a minimum frequency of 2.0% and a minimum coverage of 1,000 reads, to ensure reliable detection of minority variants.

### 2.6 Data analysis

Potential between‐farm transmission cases were defined as two or more poultry farms testing positive for LPAI virus infection of the same HA/NA subtype within a time interval between two consecutive detections of maximum 6 months. To identify statistically significant spatial clusters of infected farms, spatial cluster analysis was performed using the free software program SaTScan version 9.6 (http://www.satscan.org) (Kulldorff, 1997) for each potential between‐farm transmission case separately. Input data were represented by the background poultry farm population (variable 0), infected farms (variable 1) and geographical locations of individual farms specified as Cartesian coordinates. The Bernoulli probability model was used to scan for areas with a higher rate of infected farms than would be expected by chance (*p* < 0.05). Geographical maps were generated using the statistical software package R version 3.4.0 (R Core Team, 2017). Comparison of time intervals between virus detections and distances between infected farms was performed by using the non‐parametric Wilcoxon rank sum test, with significance defined as *p* < 0.05. Genetic analysis was performed by aligning the nucleotide consensus sequences for each gene segment separately in CLC Genomics Workbench (Qiagen). These alignments were used to calculate the nucleotide sequence identities between viruses and identify minority variants at consensus‐level variant sites.

## 3 RESULTS

### 3.1 Identification of potential between‐farm transmission cases

**Figure 1:**
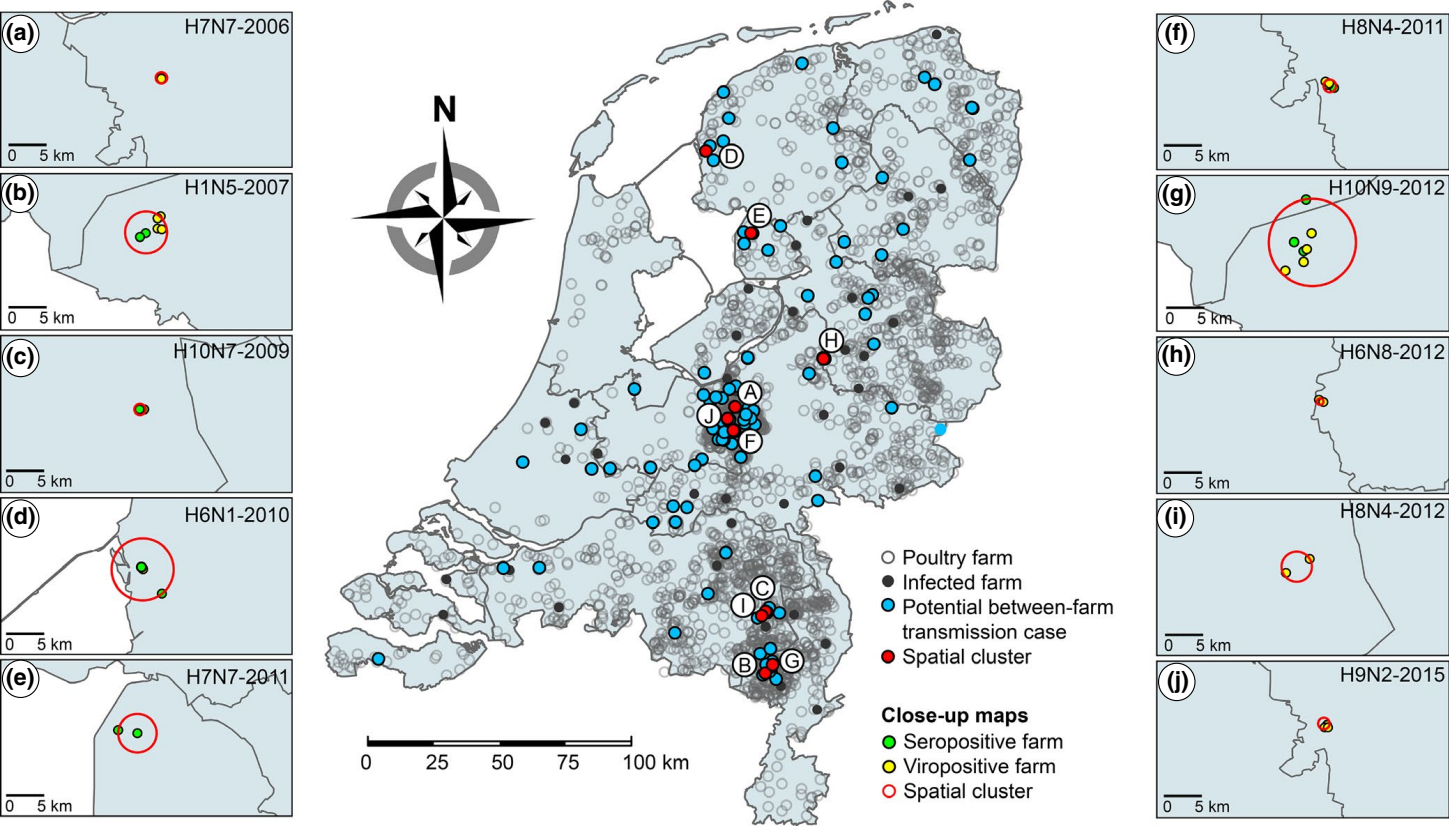
Geographical map of potential between‐farm transmission cases. Map of the Netherlands showing the geographical distribution of commercial poultry farms (open dots) (*n* = 2,379), farms infected with low pathogenic avian influenza (LPAI) virus (filled dots) (*n* = 220), farms involved in potential between‐farm transmission cases (blue) (*n* = 132) and statistically significant spatial clusters (red) (*n* = 10), including close‐up maps of 10 spatial clusters of seropositive farms (green) and viropositive farms (yellow) within potential between‐farm transmission cases H7N7‐2006 (a), H1N5‐2007 (b), H10N7‐2009 (c), H6N1‐2010 (d), H7N7‐2011 (e), H8N4‐2011 (f), H10N9‐2012 (g), H6N8‐2012 (h), H8N4‐2012 (i) and H9N2‐2015 (j). Spatial cluster analysis was performed for each potential between‐farm transmission case separately using the Bernoulli probability model (*p* < 0.05). All samples were collected as part of the national avian influenza (AI) surveillance program in the Netherlands between January 2006 and September 2016

To identify potential between‐farm transmission cases, we analysed 220 serological and virological detections of LPAI virus infections that occurred in commercial poultry in the Netherlands between 2006 and 2016, which included 162 seropositive and 58 viropositive farms. Of the virologically confirmed infections, the genome sequence of 42 LPAI viruses was obtained. Over the 10‐year surveillance period, we identified 35 potential between‐farm transmission cases involving 132 farms, including 111 seropositive and 21 viropositive farms (Figure 1; Table S2). Potential between‐farm transmission cases involved various subtypes, of which some were detected in multiple years, for example, H7N7 (2006, 2011, 2013 and 2015), H8N4 (2009, 2011, 2012, 2013 and 2015), H6N2 (2013, 2014 and 2015), H6N8 (2011, 2012 and 2013) and H9N2 (2010 and 2015). A total of 10 spatial clusters were identified (referred to as clusters A–J) (Table S3). Cluster radii ranged from 0.1 to 5.9 km, with a median radius of 1.5 km. Seven clusters included 2–3 infected farms (clusters A, C, D, E, H, I and J), and three clusters included 5–7 infected farms (clusters B, F and G). Most clusters were found in poultry‐dense areas in the southern (clusters B, C, G and I) and central (clusters A, F and J) part of the Netherlands, whereas some clusters (clusters D, E and H) were found in areas with a low poultry density. Geographical clustering of infected farms indicates potential transmission of LPAI viruses between neighbouring farms or separate introductions from the same environmental source.

### 3.2 Genetic analysis of potential between‐farm transmitted viruses

**Figure 2:**
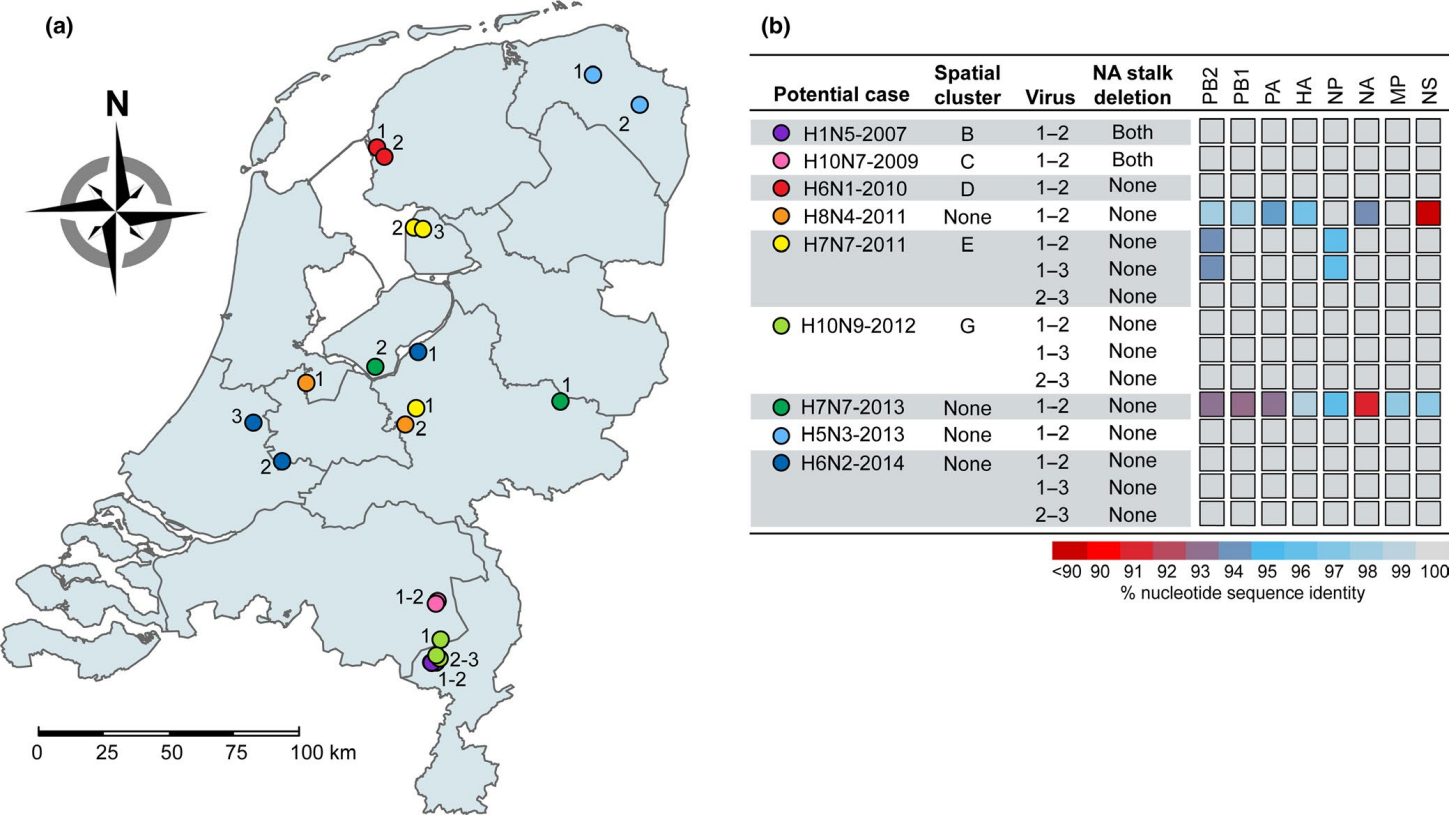
Genetic analysis of potential between‐farm transmission cases. (a) Geographical map of the Netherlands showing the collection locations of low pathogenic avian influenza (LPAI) viruses isolated from potential between‐farm transmission cases (*n* = 21). (b) Genetic analysis of LPAI viruses isolated from potential between‐farm transmission cases, showing the presence of deletions in the neuraminidase (NA) stalk region and the percentage of nucleotide sequence identity between viruses for each gene segment separately. All samples were collected as part of the national avian influenza (AI) surveillance program in the Netherlands between January 2006 and September 2016. Detailed information on the virus sequences is provided in Table S1. HA, haemagglutinin; MP, matrix protein; NA, neuraminidase; NP, nucleoprotein; NS, non‐structural protein; PA, polymerase acidic protein; PB1, polymerase basic protein 1; PB2, polymerase basic protein 2.

Next‐generation sequencing was performed to analyse potential transmissions between farms genetically. The LPAI virus sequences were obtained from 21 viropositive farms involved in nine potential between‐farm transmission cases (Table S1). In five of these cases, two or more viropositive farms were located within the same spatial cluster (clusters B, C, D, E and G). The collection locations of the 21 LPAI viruses were plotted in a geographical map of the Netherlands (Figure 2a).

After the whole genome sequences were determined, genetic analysis was performed by aligning the nucleotide consensus sequences of potentially between‐farm transmitted viruses for each gene segment separately (Figure 2b). Viruses isolated from potential cases H1N5‐2007, H10N7‐2009, H6N1‐2010, H10N9‐2012, H5N3‐2013 and H6N2‐2014 shared nucleotide sequence identities of 99.70%–100.00% in all eight gene segments. Additionally, viruses isolated from potential cases H1N5‐2007 and H10N7‐2009 both contained a deletion in the stalk region of the NA protein of 18 and 21 amino acids respectively. Viruses H7N7‐2011‐2 and H7N7‐2011‐3 also showed less than 0.3% nucleotide sequence divergence in each gene segment, whereas virus H7N7‐2011‐1 showed high sequence identities with viruses H7N7‐2011‐2 and H7N7‐2011‐3 in gene segments encoding polymerase basic protein 1 (PB1), polymerase acidic protein (PA), HA, NA, matrix protein (MP) and non‐structural protein (NS) (99.44%–100.00%), but relatively low sequence identities in gene segments encoding polymerase basic protein 2 (PB2) and nucleoprotein (NP) (93.79%–95.85%).

Low sequence identities were found between viruses isolated from potential cases H8N4‐2011 and H7N7‐2013. H8N4‐2011 viruses showed high sequence identities in NP and MP (99.60%), but relatively low sequence identities in PB2, PB1, PA, HA and NA (93.81%–98.35%). For NS, only 53.84% sequence identity was observed, demonstrating that the viruses have distinct NS alleles. H7N7‐2013 viruses shared relatively low sequence identities (<98.70%) in all gene segments, showing that the viruses were only distantly related. Altogether, whole genome sequence analysis identified highly similar viruses, sharing nucleotide sequence identities of 99.70%–100.00% in all gene segments, in seven of nine potential cases involving 16 farms. As seven farms can be considered primary infected farms, these results suggest that 9 of 21 viropositive farms may have become infected by between‐farm virus transmission.

### 3.3 Identification of minority variants

NGS was applied to detect minority variants arising from biological variation in the virus population. Minority variants were analysed for all 16 genetically closely related viruses isolated from seven potential between‐farm transmission cases using a minimum frequency of 2.0% and a minimum coverage of 1,000 reads (Table 1; Table S4). The average coverage was 4,500 reads per nucleotide position. Shared minority variants were detected at two nucleotide positions in two potential cases, that is at nucleotide position 1,017 in the HA gene of viruses H10N7‐2009‐1 and H10N7‐2009‐2 (frequencies of 3%) and nucleotide position 1,202 in the NP gene of viruses H10N9‐2012‐2 and H10N9‐2012‐3 (frequencies of 7 and 10% respectively).

**Table 1:** Minority variant analysis of genetically closely related low pathogenic avian influenza (LPAI) viruses isolated from potential between‐farm transmission cases

Potential case	Virus alignment	No. of nucleotide differences	No. of shared minority variants	No. of minority variants at consensus‐level variant sites
H1N5‐2007	1–2	4	0	4
H10N7‐2009	1–2	10	1	2
H6N1‐2010	1–2	14	0	4
H7N7‐2011	2–3	12	0	12
H10N9‐2012	1–2	25	0	4
	1–3	25	0	12
	2–3	8	1	6
H5N3‐2013	1–2	24	0	2
H6N2‐2014	1–2	9	0	4
	1–3	11	0	4
	2–3	6	0	0

Note

The number of nucleotide differences, the number of shared minority variants (i.e. minority variants that are present in both viruses) and the number of minority variants at consensus‐level variant sites (i.e. minority variants at sites that varied between viruses at consensus level) are shown. Minority variants were detected using a minimum frequency of 2.0% and a minimum coverage of 1,000 reads. All samples were collected as part of the national avian influenza (AI) surveillance program in the Netherlands between January 2006 and September 2016. Detailed information on the virus sequences is provided in Table S1.

More often, minority variants were identified at sites that differed between the consensus sequences of two aligned viruses, with frequencies ranging from 2% to 47%. In potential cases H1N5‐2007 and H7N7‐2011, minority variants were identified at all sites that varied at consensus level, making a transmission event between these farms highly plausible. In addition, a relatively high number of nucleotide variants were found in the viral subpopulation of H10N9‐2012‐3, which were present in the consensus sequence of virus H10N9‐2012‐1. In potential case H6N2‐2014, all nucleotide variants detected in the viral subpopulation of H6N2‐2014‐1 were fixed in the consensus sequence of viruses H6N2‐2014‐2 and H6N2‐2014‐3. The detection of shared minority variants and minority variants at consensus‐level variant sites provides additional indications that transmission of LPAI viruses between poultry farms occurred.

### 3.4 Epidemiological variables associated with between‐farm transmission

Genetic analysis was combined with information regarding time, distance and poultry type to identify epidemiological variables associated with between‐farm transmission (Table 2). Genetically closely related viruses were isolated within a median time interval of 8 days (range 1–36 days), which was lower but not significantly different from that of the genetically distinct viruses (median time interval of 43 days; range 6–62 days) (*p* = 0.06). The median distance between the collection locations of genetically closely related viruses was 6.3 km (range 0.6–68.9 km), which was significantly shorter compared to that of the genetically distinct viruses (median distance of 69.0 km; range 41.3–72.3 km) (*p* < 0.05). Genetically closely related viruses isolated from potential cases H1N5‐2007, H10N7‐2009, H6N1‐2010, H7N7‐2011 and H10N9‐2012 were collected within spatial clusters with distances between farms ranging from 0.6 to 6.9 km, suggesting local spread between farms or independent infections by the same local source. Additionally, seropositive farms were detected within the same spatial cluster in potential cases H1N5‐2007, H6N1‐2010 and H10N9‐2012, indicating more farms were infected with the same virus. Interestingly, in potential cases H1N5‐2007 and H10N9‐2012, two infected farms within the same spatial cluster shared the same owner. Farms involved in potential cases H5N3‐2013 and H6N2‐2014 were located at, respectively, 21.3 and 18.5–68.9 km distance, indicating long‐distance spread.

**Table 2:** Epidemiological information on low pathogenic avian influenza (LPAI) viruses isolated from potential between‐farm transmission cases

Potential between‐farm case	Virus alignment	Genetic relationship^a^	Time interval (days)	Distance (km)	Spatial cluster (*p* < 0.05)^b^	No. of infected farms within cluster	Cluster radius (km)	Poultry type
H1N5‐2007	1–2	Close	22	0.9	B	6	3.0	Tu‐Tu
H10N7‐2009	1–2	Close	1	0.6	C	2	0.3	Tu‐Oc
H6N1‐2010	1–2	Close	28	4.5	D	3	4.4	Ic‐Ic
H8N4‐2011	1–2	Distant	62	41.3	None	n/a	n/a	Oc‐Oc
H7N7‐2011	1–2	Distant	42	69.2	E	2	2.7	Oc‐Oc
	1–3	Distant	44	68.7				Oc‐Oc
	2–3	Close	2	3.4				Oc‐Tu
H10N9‐2012	1–2	Close	28	6.3	G	7	5.9	Oc‐Tu
	1–3	Close	36	6.9				Oc‐Tu
	2–3	Close	8	1.3				Tu‐Tu
H7N7‐2013	1–2	Distant	6	72.3	None	n/a	n/a	Oc‐Oc
H5N3‐2013	1–2	Close	11	21.3	None	n/a	n/a	Oc‐Oc
H6N2‐2014	1–2	Close	5	18.5	None	n/a	n/a	Du‐Oc
	1–3	Close	8	68.9				Du‐Du
	2–3	Close	3	67.1				Oc‐Du

Note

The time interval between virus detections, the distance between infected farms, the presence of statistically significant spatial clusters and poultry types are shown. All samples were collected as part of the national avian influenza (AI) surveillance program in the Netherlands between January 2006 and September 2016. Detailed information on the virus sequences is provided in Table S1.

Abbreviations: Du: domestic duck; Ic: indoor layer chicken; n/a: not applicable; Oc: outdoor layer chicken; Tu: turkey.

a The genetic relationship was considered ‘close’ if the viruses share nucleotide sequence identities of ≥99.70% in all gene segments. The genetic relationship was considered ‘distant’ if the viruses share a nucleotide sequence identity of <99.70% in at least one gene segment.

b The capital letters refer to close‐up maps of spatial clusters presented in Figure 1.

Finally, poultry types involved in potential between‐farm transmission cases were examined. All genetically distinct viruses were isolated from outdoor chicken layer farms. The 16 genetically closely related viruses were isolated from six chicken layer farms with outdoor facilities (38%) and 10 farms with an indoor housing system, including six turkey farms (38%), two duck farms (13%) and two chicken layer farms (13%). Potential spread within a poultry type was observed between farms infected with viruses H1N5‐2007‐1 and H1N5‐2007‐2 (turkeys), H6N1‐2010‐1 and H6N1‐2010‐2 (indoor chickens), H10N9‐2012‐2 and H10N9‐2012‐3 (turkeys), H5N3‐2013‐1 and H5N3‐2013‐2 (outdoor chickens) and H6N2‐2014‐1 and H6N2‐2014‐3 (domestic ducks). Within the spatial clusters of potential cases H1N5‐2007 and H6N1‐2010, the seropositive farms were of the same poultry type, and no infections were detected in farms holding a different poultry type (Table S3). In contrast, potential spread between farms holding different poultry types was observed in farms infected with viruses H10N7‐2009‐1 and H10N7‐2009‐2 (turkeys–outdoor chickens), H7N7‐2011‐1 and H7N7‐2011‐2 (outdoor chickens–turkeys), H10N9‐2012‐1 and the two other infected farms (outdoor chickens–turkeys) and H6N2‐2014‐2 and the two other infected farms (outdoor chickens–domestic ducks). The combined results suggest that between‐farm transmission of LPAI viruses was not related to indoor or outdoor housing systems and not restricted to holdings of the same poultry type.

## 4 DISCUSSION

This study evaluates the contribution of between‐farm transmission to the overall incidence of LPAI virus infections in commercial poultry in the Netherlands. We analysed serological and virological detections of LPAI virus infections to identify potential between‐farm transmission events. Subsequently, genetic analysis was combined with spatio‐temporal and poultry type information to identify epidemiological variables associated with between‐farm transmission. Over a 10‐year surveillance period, we identified 35 potential between‐farm transmission cases involving 132 of 220 infected poultry farms. We showed that in 10 of these cases farms clustered geographically. The number of farms involved in each case was relatively small (2–7 infected farms), as compared to previous LPAI virus outbreaks, including those of subtypes H7N2 (1996–1998 and 2001–2002) in the United States, and H7N1 (1999 and 2000–2001), H7N3 (2002–2003 and 2004) and H5N2 (2010–2012) in Italy, that reported between 24 and 388 infected farms (Capua & Alexander, 2004; Mughini‐Gras et al., 2014). Some subtypes were detected in multiple years, which may be due to recurrent virus introductions from the wild bird population. However, no related wild bird viruses were detected in the same time frame between 2006 and 2011 (Verhagen et al., 2017), or in recent years. Also, none of the viruses were associated with HPAI outbreaks that were reported in the Netherlands in 2014, 2016 and 2017 (Beerens et al., 2017, 2018; Bouwstra et al., 2015).

Genetic analysis was performed using the whole genome sequences of 21 LPAI viruses isolated from nine potential cases. This analysis revealed that viruses isolated from potential cases H8N4‐2011 and H7N7‐2013 were only distantly related. Between‐farm transmission could therefore be excluded in these two cases. In addition, virus H7N7‐2011‐1 showed low sequence identities with viruses H7N7‐2011‐2 and H7N7‐2011‐3 in two gene segments, which is presumably due to reassortment of gene segments between co‐circulating viruses. Reassortment events are commonly observed in wild birds, the natural host of a vast diversity of AI viruses (Macken, Webby, & Bruno, 2006). Therefore, reassortment likely occurred in the wild bird population and two distinct reassortment variants were subsequently introduced into the poultry facilities separately.

Genetically closely related viruses, showing less than 0.3% nucleotide sequence divergence in each gene segment, were identified in seven of nine virologically analysed cases involving 16 poultry farms. The close genetic relationship between the viruses suggests between‐farm transmission or separate introductions from the same environmental source. Neuraminidase stalk deletions were identified in potential cases H1N5‐2007 and H10N7‐2009. A deletion in the NA stalk region is considered a marker of virus adaptation to chickens, turkeys and other gallinaceous hosts (Li & Cardona, 2010; Li, Zu Dohna, Cardona, Miller, & Carpenter, 2011), and is rarely detected in wild birds without a link to poultry. Neuraminidase stalk deletions cause a change in tropism from the intestine to the respiratory tract in chickens (Hoffmann et al., 2012; Sorrell, Song, Pena, & Perez, 2010), thereby increasing virus pathogenicity (Munier et al., 2010). The length and position of NA stalk deletions are highly variable (Li et al., 2011). The fact that NA stalk deletions of identical length and position were identified strongly indicates that between‐farm transmission occurred.

Moreover, shared minority variants or minority variants at consensus‐level variant sites were identified in all seven potential between‐farm transmission cases of genetically closely related viruses. These minority variants, although often present at low level, suggest that the viruses are genetically more closely related than predicted based on the consensus sequence. Interestingly, genetically closely related viruses isolated from potential cases H1N5‐2007 and H7N7‐2011 showed minority variants at all consensus‐level variant sites. In these two cases, the virus on the secondary infected farm was likely isolated shortly after direct transmission from the primary infected farm or introduction from the same environmental source. In contrast, a relatively low number of minority variants together with a relatively high number of nucleotide differences in potential case H5N3‐2013 suggest prolonged within‐flock transmission before samples were collected. In some cases, the genetic relationship based on minority variants may be underestimated due to passaging of the virus strains in eggs.

Surprisingly, minority variant analysis indicated that virus H10N9‐2012‐1 was genetically more closely related to virus H10N9‐2012‐3 than to virus H10N9‐2012‐2, despite the larger time interval between the collection dates. At the same time, viruses H10N9‐2012‐1 and H10N9‐2012‐2 shared two fixed nucleotide variants that were not present in virus H10N9‐2012‐3. We therefore hypothesize that the virus was transmitted from H10N9‐2012‐1 to H10N9‐2012‐2 and H10N9‐2012‐3 via another (seropositive) farm within the same spatial cluster. This hypothesis is supported by the relatively high number of nucleotide differences between virus H10N9‐2012‐1 and the other two isolates. Minority variant analysis also revealed that viruses H6N2‐2014‐1 and H6N2‐2014‐2 shared four fixed nucleotide variants that were not present in virus H6N2‐2014‐3, and viruses H6N2‐2014‐1 and H6N2‐2014‐3 shared two fixed nucleotide variants that were not present in virus H6N2‐2014‐2. No minority variants were identified at sites that differed between H6N2‐2014‐2 and H6N2‐2014‐3. Based on these results, we assume that virus H6N2‐2014‐1 acted as a precursor virus for both viruses H6N2‐2014‐2 and H6N2‐2014‐3.

Contact tracing to study the intensity of movements between farms could reveal potential modes of transmission, but is generally not performed for non‐notifiable LPAI viruses. Here, we analysed other epidemiological links between farms, such as the time interval between virus detections, the distance between farms and poultry types, to identify variables that may be associated with between‐farm transmission.

Temporal analysis demonstrated that genetically closely related viruses were detected within a median time interval of 8 days (range 1–36 days). Previous studies have shown that viral shedding can already be observed from one day after experimental infection in chickens (Swayne & Beck, 2005; van der Goot, de Jong, Koch, & Van Boven, 2003). The mean infectious period of individual LPAI virus‐infected birds was estimated to range between 4 and 8 days (Comin, Klinkenberg, Marangon, Toffan, & Stegeman, 2011; Gonzales, van der Goot, Stegeman, Elbers, & Koch, 2011; van der Goot et al., 2003). However, the duration of the infectious period of an infected flock can take much longer, depending on within‐flock transmission dynamics influenced by the virus and flock characteristics, such as poultry type, age of production and the presence of concomitant diseases (Gonzales et al., 2012). At flock level, the infectious period is estimated to range between 1 and 2 months for chickens (Gonzales, Boender, Elbers, Stegeman, & de Koeijer, 2014), and 2 and 11 months for turkeys (Comin et al., 2011). This is much longer compared to HPAI, as most LPAI infections remain subclinical and control measures are not applied for subtypes other than H5 and H7. The time intervals between the potential cases fall within the estimated infectious period of LPAI virus‐infected flocks and are therefore consistent with between‐farm transmission.

Our study further suggests that both local and long‐distance transmissions occurred and that between‐farm transmission was not restricted to holdings of the same poultry type. Additionally, no relation was found between indoor or outdoor housing systems and potential between‐farm transmission. However, despite representing only 2% of the total poultry population in the Netherlands, turkeys were involved in a relatively high number of potential between‐transmission cases. This may be explained by a higher susceptibility of this species to AI viruses (Pillai, Pantin‐Jackwood, Yassine, Saif, & Lee, 2010). Interestingly, all genetically distinct viruses were isolated from outdoor chickens, which may become infected more easily through direct or indirect contact with wild birds (Koch & Elbers, 2006).

Potential local spread within a poultry type was observed between farms infected with viruses H1N5‐2007‐1 and H1N5‐2007‐2, H6N1‐2010‐1 and H6N1‐2010‐2 and H10N9‐2012‐2 and H10N9‐2012‐3. During these events, transmission may have occurred via movement of persons or contaminated equipment between neighbouring farms, which is likely to occur between farms of the same poultry type because of a high probability of shared personnel, equipment and transport services (Leibler et al., 2010; McQuiston et al., 2005; Thomas et al., 2005; Vieira et al., 2009). This transmission route is supported by the fact that no influenza infections were detected in farms holding a different poultry type in potential cases H1N5‐2007 and H6N1‐2010, and two infected farms in potential cases H1N5‐2007 and H10N9‐2012 shared the same owner. Since AI viruses can persist for extended periods in the environment (Brown et al., 2009; Thompson & Bennett, 2017), transport of contaminated materials is also considered an important route of virus spread over long distances (Mulatti et al., 2010). Between‐farm transmission via human‐mediated transport was therefore considered the most probable route of transmission for potential long‐distance spread within a poultry type, which was observed between farms infected with viruses H5N3‐2013‐1 and H5N3‐2013‐2, and H6N2‐2014‐1 and H6N2‐2014‐3.

Alternatively, virus may have been transmitted between neighbouring farms by vectors (Velkers et al., 2017; Wanaratana et al., 2013) or via airborne transmission when virus particles or virus‐contaminated dust particles are being dispersed into the environment (Jonges et al., 2015; Spekreijse et al., 2013). These transmission routes may explain potential local spread between farms holding different poultry types, which were observed between farms infected with viruses H10N7‐2009‐1 and H10N7‐2009‐2, and H7N7‐2011‐2 and H7N7‐2011‐3, and from H10N9‐2012‐1 to the other infected farms. During the latter event, virus was detected in air samples up to 60 metres downwind of two infected turkey farms 2–9 days after infection was confirmed (Jonges et al., 2015). However, detection decreased rapidly with distance. Hence, the probability of between‐flock transmission by air decreases with increasing distance (Boender et al., 2007; Ssematimba, Hagenaars, & de Jong, 2012) and will depend heavily on environmental conditions, such as wind (Ypma et al., 2013). In addition, most potential transmission events occurred between farms with indoor facilities, suggesting airborne transmission is less likely because of mechanical barriers.

In conclusion, our study indicates that between‐farm transmission occurred in seven of nine virologically analysed cases. Based on these findings, transmission between poultry farms likely contributes considerably to the incidence of LPAI virus infections in poultry, although separate introductions from the wild bird reservoir cannot be excluded. In this study, genetic analysis was limited to few potential between‐farm transmission cases for which virus was isolated. More frequent collection of samples for virological monitoring of non‐notifiable LPAI viruses in poultry would be of great value to obtain more knowledge on LPAI virus transmission dynamics. This study highlights the value of genetic analysis to complement serological data and to improve epidemiological investigations on LPAI virus transmissions, which can be used to guide disease control strategies.

## CONFLICT OF INTEREST

The authors declare no conflict of interest.
