# Supplementary material for: Genetic analysis identifies potential transmission of low pathogenic avian influenza viruses between poultry farms
Source: Transbound Emerg Dis. 2019 Apr 25;66(4):1653–64. doi: 10.1111/tbed.13199 (PMC6850361; doi:10.1111/tbed.13199)
Supplement: Supplementary file 1 [file TBED-66-1653-s001.pdf]

S1 Table. Viruses isolated from potential between-farm transmission cases. Detailed information on low pathogenic avian influenza (LPAI) viruses isolated from potential between-farm transmission cases. All viruses were detected as part of the national avian influenza (AI) surveillance program in the Netherlands between January 2006 and September 2016. Whole genome consensus sequences were submitted to the GISAID's EpiFlu™ Database (https://www.gisaid.org).

Potential case	Isolate number and name	Poultry type	No. of animals per farm	Farm culled	Passage details	Collection location (province)	Collection date	GISAID isolate ID
H1N5-2007	1. A/Turkey/Netherlands/07014290/2007	Turkey	n/a	No	E2	Limburg	2007-05-31	EPI_ISL_309820
	2. A/Turkey/Netherlands/07016245/2007	Turkey	19,000	No	E1	Limburg	2007-06-22	EPI_ISL_309821
H10N7-2009	1. A/Turkey/Netherlands/09006938/2009	Turkey	11,500	No	E1	Noord-Brabant	2009-04-14	EPI_ISL_309822
	2. A/Chicken/Netherlands/09006942/2009	Outdoor layer chicken	8,500	No	E1	Noord-Brabant	2009-04-15	EPI_ISL_309798
H6N1-2010	1. A/Chicken/Netherlands/10010413/2010	Indoor layer chicken	25,500	No	E1	Friesland	2010-06-21	EPI_ISL_309801
	2. A/Chicken/Netherlands/10012103/2010	Indoor layer chicken	43,000	No	E1	Friesland	2010-07-19	EPI_ISL_309802
H8N4-2011	1. A/Chicken/Netherlands/11004004/2011	Outdoor layer chicken	n/a	No	E2	Utrecht	2011-03-09	EPI_ISL_309804
	2. A/Chicken/Netherlands/11008325/2011	Outdoor layer chicken	n/a	No	Original	Gelderland	2011-05-10	EPI_ISL_309806
H7N7-2011	1. A/Chicken/Netherlands/11008327/2011	Outdoor layer chicken	9,000	Yes	E2	Gelderland	2011-05-12	EPI_ISL_309929
	2. A/Chicken/Netherlands/11011392/2011	Outdoor layer chicken	47,000	Yes	E2	Flevoland	2011-06-23	EPI_ISL_309808
	3. A/Turkey/Netherlands/11011530/2011	Turkey	7,000	Yes	E2	Flevoland	2011-06-25	EPI_ISL_309823
H10N9-2012	1. A/Chicken/Netherlands/12002495-001-005/2012	Outdoor layer chicken	22,000	No	E1	Noord-Brabant	2012-02-06	EPI_ISL_309809
	2. A/Turkey/Netherlands/12004763-001-004/2012	Turkey	4,000	No	E1	Limburg	2012-03-05	EPI_ISL_309825
	3. A/Turkey/Netherlands/12005615/2012	Turkey	33,000	No	E1	Limburg	2012-03-13	EPI_ISL_309826
H7N7-2013	1. A/Chicken/Netherlands/13003601/2013	Outdoor layer chicken	85,000	Yes	Original	Gelderland	2013-03-12	EPI_ISL_309811
	2. A/Chicken/Netherlands/13003983/2013	Outdoor layer chicken	24,000	Yes	Original	Flevoland	2013-03-18	EPI_ISL_309812
H5N3-2013	1. A/Chicken/Netherlands/13015884/2013	Outdoor layer chicken	10,000	Yes	Original	Groningen	2013-11-29	EPI_ISL_309813
	2. A/Chicken/Netherlands/13016263-031-035/2013	Outdoor layer chicken	12,000	Yes	Original	Groningen	2013-12-10	EPI_ISL_309814
H6N2-2014	1. A/Duck/Netherlands/14015610/2014	Duck	n/a	No	Original	Utrecht	2014-11-17	EPI_ISL_309833
	2. A/Chicken/Netherlands/14016059/2014	Outdoor layer chicken	n/a	No	Original	Zuid-Holland	2014-11-22	EPI_ISL_309930
	3. A/Duck/Netherlands/14016396/2014	Duck	12,000	No	Original	Gelderland	2014-11-25	EPI_ISL_309835
