# Supplementary material for: Genetic analysis identifies potential transmission of low pathogenic avian influenza viruses between poultry farms
Source: Transbound Emerg Dis. 2019 Apr 25;66(4):1653–64. doi: 10.1111/tbed.13199 (PMC6850361; doi:10.1111/tbed.13199)
Supplement: Supplementary file 2 [file TBED-66-1653-s002.pdf]

S2 Table. Identification of potential between-farm transmission cases. Identification of potential between-farm transmission cases of low pathogenic avian influenza (LPAI) viruses based on serological and virological surveillance data. Potential between-farm transmission cases were defined as two or more poultry farms testing positive for LPAI virus infection of the same hemagglutinin (HA) and neuraminidase (NA) subtype combination within a time interval between two consecutive detections of maximum six months. All samples were collected as part of the national avian influenza (AI) surveillance program in the Netherlands between January 2006 and September 2016.

Potential case	Collection date first detection	Collection date last detection	Time span (days)	Total no. of infected farms	No. of infected chicken farms	No. of infected turkey farms	No. of infected duck farms	No. of seropositive farms	No. of viropositive farms
H7N7-2006	2006-07-26	2006-08-03	8	2	1			2	
H1N5-2007	2007-06-01	2007-08-21	81	6		6		4	2
H10N7-2009	2009-04-14	2009-04-15	1	2	1	1			2
H8N4-2009	2009-08-10	2010-07-06	330	6	6			6	
H6N1-2010	2010-05-25	2010-07-15	51	3	3			1	2
H7N4-2010	2010-05-14	2010-05-18	4	2	2			2	
H9N2-2010	2010-11-12	2013-03-12	851	12	11			12	
H6N8-2011	2011-01-19	2011-02-28	40	2	2			2	
H7N7-2011	2011-02-04	2011-06-26	142	4	2	2		1	3
H8N4-2011	2011-03-10	2011-10-06	210	9	7		2	7	2
H1N1-2011	2011-05-31	2011-07-12	42	2	1		1	2	
H10N9-2012	2012-02-07	2012-04-09	62	7	2	5		4	3
H2N3-2012	2012-02-20	2012-08-13	175	2	2			2	
H6N8-2012	2012-03-02	2012-05-28	87	4	4			4	
H2N9-2012	2012-04-03	2012-09-25	175	4	4			4	
H8N4-2012	2012-09-20	2013-01-19	121	2		2		2	
H11N9-2012	2012-12-20	2013-02-05	47	3	3			3	
H7N7-2013	2013-03-11	2013-06-13	94	3	3			1	2
H6N2-2013	2013-05-06	2013-06-20	45	2	2			2	
H7N1-2013	2013-05-31	2013-06-17	17	2	2			2	
H5N2-2013	2013-09-09	2014-03-31	203	3	2		1	3	
H8N4-2013	2013-09-23	2014-09-18	360	6	6			6	
H11N9-2013	2013-10-14	2013-10-14	0	2	2			2	

H5N3-2013	2013-11-28	2014-03-31	123	4	4			2	2
H6N8-2013	2013-11-29	2014-04-01	123	3	2	1		3	
H9N5-2013	2013-11-11	2014-01-03	53	3	3			3	
H2N7-2014	2014-02-27	2014-09-18	203	3	3			3	
H6N2-2014	2014-03-13	2015-02-23	347	7	4	1	2	4	3
H5N2-2014	2014-11-22	2015-04-02	131	2	2			2	
H2N5-2015	2015-01-05	2015-07-06	182	3	3			3	
H9N2-2015	2015-02-12	2016-03-07	389	8	7		1	8	
H6N2-2015	2015-11-12	2016-03-28	137	2	2			2	
H7N7-2015	2015-03-10	2015-06-22	104	3	3			3	
H8N4-2015	2015-06-23	2015-06-25	2	2	2			2	
H9N7-2016	2016-01-14	2016-03-07	53	2	1			2	
**Total**				132	104	18	7	111	21
