# Supplementary material for: Genetic analysis identifies potential transmission of low pathogenic avian influenza viruses between poultry farms
Source: Transbound Emerg Dis. 2019 Apr 25;66(4):1653–64. doi: 10.1111/tbed.13199 (PMC6850361; doi:10.1111/tbed.13199)
Supplement: Supplementary file 3 [file TBED-66-1653-s003.pdf]

S3 Table. Spatial clusters within potential between-farm transmission cases. Total number of farms and number of infected farms located within spatial clusters per poultry type. Spatial cluster analysis was performed for each potential between-farm transmission case separately using the Bernoulli probability model ($p<0.05$). All samples were collected as part of the national AI surveillance program in the Netherlands between January 2006 and September 2016.

Potential case	Spatial cluster ($p<0.05$) †	Cluster radius (km)	Total no. of farms	Total no. of chicken farms	Total no. of turkey farms	Total no. of duck farms	No. of infected farms	No. of infected chicken farms	No. of infected turkey farms	No. of infected duck farms	No. of seropositive farms	No. of viropositive farms
H7N7-2006	A	0.1	3	3			2	2			2	
H1N5-2007	B	3.0	25	16	9		6		6		4	2
H10N7-2009	C	0.3	4	3	1		2	1	1			2
H6N1-2010	D	4.4	5	4		1	3	3			1	2
H7N7-2011	E	2.7	3	2	1		2	1	1			2
H8N4-2011	F	0.9	10	9		1	5	4		1	4	1
H10N9-2012	G	5.9	90	75	14	1	7	2	5		4	3
H6N8-2012	H	0.5	2	2			2	2			2	
H8N4-2012	I	2.1	5	3	2		2		2		2	
H9N2-2015	J	0.8	8	8			3	3			3	
**Total**			155	125	27	3	34	18	15	1	22	12

† The capital letters refer to close-up maps of spatial clusters presented in Fig 1.
