# Supplementary material for: Genetic analysis identifies potential transmission of low pathogenic avian influenza viruses between poultry farms
Source: Transbound Emerg Dis. 2019 Apr 25;66(4):1653–64. doi: 10.1111/tbed.13199 (PMC6850361; doi:10.1111/tbed.13199)
Supplement: Supplementary file 4 [file TBED-66-1653-s004.pdf]

S4 Table. Identification of minority variants. Minority variant analysis of genetically closely related low pathogenic avian influenza (LPAI) viruses isolated from potential between-farm transmission cases. All nucleotide positions that varied between viruses at consensus level are shown. Minority variants consist of shared minority variants (i.e. minority variants that are present in both viruses) and minority variants at consensus-level variant sites (i.e. minority variants at sites that varied between viruses at consensus level). Minority variants were detected using a minimum frequency of 2.0% and a minimum coverage of 1,000 reads. All viruses were detected as part of the national avian influenza (AI) surveillance program in the Netherlands between January 2006 and September 2016. Detailed information on the virus sequences is provided in S1 Table.

					Virus 1		Virus 2		Virus 3	
**Potential case**	**Gene segment †**	**Nucleotide position**	**Variant A**	**Variant B**	**Variant A**	**Variant B**	**Variant A**	**Variant B**	**Variant A**	**Variant B**
H1N5-2007	PB2	2049	T	C	100%	0%	15%	85%		
	PA	885	A	C	67%	33%	0%	100%		
	HA	523	G	A	61%	39%	0%	100%		
	NS	521	C	T	65%	35%	0%	100%		
H10N7-2009	PB2	2061	A	G	100%	0%	26%	74%		
	PB2	2065	G	A	100%	0%	0%	100%		
	PB1	472	G	A	100%	0%	0%	100%		
	PB1	1930	G	A	100%	0%	0%	100%		
	PB1	2142	A	G	100%	0%	0%	100%		
	PA	2059	A	C	100%	0%	0%	100%		
	HA	766	C	T	100%	0%	11%	89%		
	HA	1017	T	A	97%	3%	97%	3%		
	NP	337	G	A	100%	0%	0%	100%		
	NS	30	G	A	100%	0%	0%	100%		
	NS	284	A	G	100%	0%	0%	100%		
H6N1-2010	PB2	646	C	A	100%	0%	0%	100%		
	PB2	2187	G	A	100%	0%	0%	100%		
	PB1	202	T	C	100%	0%	0%	100%		
	PA	73	A	G	100%	0%	0%	100%		
	PA	1053	T	C	100%	0%	0%	100%		
	PA	1071	A	T	100%	0%	0%	100%		
	PA	1133	T	G	100%	0%	0%	100%		
	PA	1892	G	T	100%	0%	0%	100%		
	NP	287	C	T	100%	0%	0%	100%		
	NA	284	C	T	100%	0%	2%	98%		
	NA	444	A	C	55%	45%	0%	100%		
	NA	710	G	A	57%	43%	0%	100%		
	NA	1297	G	A	100%	0%	0%	100%		
	NS	214	G	A	100%	0%	4%	96%		

H7N7-2011	PB2	232	A	C	100%	0%	16%	84%		
	PB2	513	C	T	100%	0%	16%	84%		
	PB2	1084	C	T	100%	0%	16%	84%		
	PB2	1359	C	T	100%	0%	16%	84%		
	PB1	1011	T	C	100%	0%	15%	85%		
	PA	497	T	C	73%	27%	10%	90%		
	PA	1842	G	A	100%	0%	15%	85%		
	HA	445	G	A	100%	0%	32%	68%		
	HA	629	A	G	72%	28%	8%	92%		
	HA	1238	C	A	100%	0%	35%	65%		
	NA	631	T	C	100%	0%	12%	88%		
	NA	926	G	A	100%	0%	12%	88%		
H5N3-2013	PB2	260	G	A	100%	0%	0%	100%		
	PB2	702	A	G	100%	0%	0%	100%		
	PB2	741	A	C	100%	0%	0%	100%		
	PB1	213	C	T	100%	0%	0%	100%		
	PB1	456	A	G	100%	0%	0%	100%		
	PB1	578	A	G	100%	0%	0%	100%		
	PB1	1158	C	T	100%	0%	12%	88%		
	PB1	1611	C	T	100%	0%	0%	100%		
	PB1	1654	A	C	100%	0%	0%	100%		
	PB1	1869	T	C	100%	0%	0%	100%		
	PA	294	T	C	100%	0%	0%	100%		
	PA	1158	G	T	100%	0%	0%	100%		
	HA	460	T	-	100%	0%	0%	100%		
	HA	461	A	-	100%	0%	0%	100%		
	HA	462	T	-	100%	0%	0%	100%		
	HA	464	A	T	100%	0%	0%	100%		
	HA	606	A	G	100%	0%	0%	100%		
	HA	617	A	T	100%	0%	0%	100%		
	HA	859	G	A	100%	0%	0%	100%		
	HA	1077	C	T	100%	0%	0%	100%		
	NP	249	A	G	100%	0%	0%	100%		
	NP	260	A	G	100%	0%	0%	100%		
	NA	1143	A	G	100%	0%	0%	100%		
	MP	932	T	G	95%	5%	0%	100%		
H10N9-2012	PB2	590	A	C	100%	0%	32%	68%	100%	0%
	PB2	1048	C	T	100%	0%	32%	68%	100%	0%
	PB2	1960	G	A	100%	0%	0%	100%	3%	97%
	PB2	2300	T	G	100%	0%	0%	100%	0%	100%
	PB1	318	C	A	100%	0%	100%	0%	4%	96%
	PB1	1383	G	A	100%	0%	0%	100%	7%	93%

	PB1	1908	C	T	100%	0%	0%	100%	6%	94%
	PB1	2218	A	G	100%	0%	0%	100%	0%	100%
	PA	392	T	C	100%	0%	0%	100%	3%	97%
	PA	666	T	C	100%	0%	32%	68%	100%	0%
	PA	1284	A	G	100%	0%	0%	100%	6%	94%
	PA	1355	C	T	100%	0%	100%	0%	6%	94%
	PA	1524	C	T	100%	0%	0%	100%	6%	94%
	PA	1864	A	G	100%	0%	0%	100%	3%	97%
	HA	49	G	A	100%	0%	100%	0%	0%	100%
	HA	544	A	G	100%	0%	30%	70%	100%	0%
	HA	566	C	A	100%	0%	0%	100%	0%	100%
	HA	769	T	C	100%	0%	0%	100%	0%	100%
	HA	1379	T	C	100%	0%	0%	100%	0%	100%
	NP	681	G	A	100%	0%	0%	100%	3%	97%
	NP	939	T	C	100%	0%	0%	100%	3%	97%
	NP	951	G	A	100%	0%	0%	100%	3%	97%
	NP	1090	A	G	100%	0%	0%	100%	0%	100%
	NP	1202	C	G	100%	0%	93%	7%	90%	10%
	NA	45	T	C	100%	0%	0%	100%	0%	100%
	NA	48	T	C	100%	0%	0%	100%	0%	100%
	NA	1054	G	A	100%	0%	100%	0%	0%	100%
	MP	421	A	G	100%	0%	0%	100%	0%	100%
	NS	209	A	G	100%	0%	0%	100%	0%	100%
	NS	338	G	A	100%	0%	0%	100%	0%	100%
H6N2-2014	PB2	1785	A	C	79%	21%	0%	100%	0%	100%
	PB1	239	G	A	64%	36%	0%	100%	0%	100%
	PB1	239	G	A	64%	36%	0%	100%	0%	100%
	PB1	354	T	C	100%	0%	0%	100%	0%	100%
	PB1	1833	G	A	78%	22%	0%	100%	0%	100%
	PB1	1836	G	A	100%	0%	0%	100%	0%	100%
	PA	318	A	G	100%	0%	100%	0%	0%	100%
	HA	236	A	G	100%	0%	100%	0%	0%	100%
	HA	376	G	T	100%	0%	0%	100%	100%	0%
	NP	46	A	G	53%	47%	0%	100%	0%	100%
	NA	45	T	C	100%	0%	100%	0%	0%	100%
	NA	558	A	G	100%	0%	0%	100%	100%	0%
	MP	604	A	G	100%	0%	0%	100%	0%	100%
	NS	430	A	G	100%	0%	100%	0%	0%	100%

† PB2, polymerase basic protein 2; PB1, polymerase basic protein 1; PA, polymerase acidic protein; NP, nucleoprotein; HA, hemagglutinin; NP, nucleoprotein; NA, neuraminidase; MP, matrix protein; NS, nonstructural protein.
